# Impact of window design on the lighting environment of GAP-certified naturally illuminated broiler houses

**DOI:** 10.1371/journal.pone.0325420

**Published:** 2025-06-04

**Authors:** Joshua A. Etherton, John E. Linhoss, Jeremiah D. Davis, Joseph L. Purswell, Jessica D. Starkey

**Affiliations:** 1 Department of Biosystems Engineering, Auburn University, Auburn, Alabama, United States of America; 2 National Poultry Technology Center, Auburn University, Auburn, Alabama, United States of America; 3 United States Department of Agricultural Research Service Poultry Research Unit, Starkville, Mississippi, United States of America; 4 Department of Poultry Science, Auburn University, Auburn, Alabama, United States of America; Ain Shams University Faculty of Agriculture, EGYPT

## Abstract

Recent changes in consumers’ desire for alternative rearing programs have prompted integrators to adopt varying fenestration designs in commercial broiler houses, most notably the inclusion of natural light (NL) via windows. The objectives of this study were to compare light intensity, spatial distribution, and uniformity in two 18.2 × 182.9 m commercial broiler houses in southeast Alabama with different window designs. Window designs in both houses met Global Animal Partnership (GAP) NL standards. The one-sided window (1SW) design had 23 translucent windows (1.42 × 1.09 m) that were all located on the north wall. The two-sided window (2SW) design had 58 translucent windows (0.95 × 0.60 m) located on both the north and south sidewalls and two additional windows of the same size on the west end wall (brooding end). Data acquisition systems were constructed to collect floor light intensity at 750 locations per replicate in both houses. Two replicates were collected for tunnel and brooding conditions in each house at solar noon ± 1 h. Mean light intensity in three house sections (fan, mid, and pad) were compared as well as whole house data for both tunnel and brood conditions. The GSTAT package in R was used to spatially map light intensities. During tunnel ventilation conditions, mean light intensity values were 1.8 times and 6.5 times higher in the mid and pad sections, respectively, in the 2SW design than the 1SW design. Light intensities during brood conditions were similar between designs (1SW = 44.7 lx; 2SW = 43.7 lx) due to the masking effect of the brighter artificial lighting targets (brooding = 43 lx; tunnel = 1 lx). Coefficients of variation (CV) were higher in the 1SW than the 2SW during brooding [63.7% (1SW) vs 56.4% (2SW)] and tunnel [192.2% (1SW) vs 143.9% (2SW)], indicating reduced spatial uniformity in the 1SW house. This study showed that the 2SW design can lead to higher overall intensities and improved spatial uniformity during tunnel conditions. Results from this study could help inform future window designs in commercial broiler houses.

## Introduction

In some commercial broiler industries artificial lighting programs are used as a means of regulating broiler behavior and activity. In the U.S., artificial lighting programs became increasingly important as growers began transitioning from curtain-sided houses to solid-sidewall houses in the late 1990s, which gave the industry much more control over the light environment [1]. However, recent changes in consumer preferences have caused some integrators to shift towards the use of alternative rearing programs such as the Global Animal Partnership (GAP). GAP is one of the most common alternative rearing programs in the U.S. and requires the provision of NL in commercial broiler houses for some of its standards. Installing windows into houses to allow for NL is a deviation from the industry norms and its effects on the lighting environment and broiler response are not completely understood.

Research studying light intensity and spatial uniformity in commercial broiler houses has become more prevalent. The most striking result from these studies is the influence that operating tunnel fans have on the lighting environment. Studies in the U.S. and Brazil have found that light ingress from tunnel ventilation fans can result in intensities of 75 lx to 4,450 lx [2,3]. These intensities are much higher than the commercial growout targets in the U.S., which are often well below 5 lx [4]. In addition, studies have reported that 10–50% of the total floor area can be influenced by light ingress from tunnel ventilation fans [5,6]. Fan shades can reduce light intrusion in the fan section by as much as 93.8%, however, fan shades are not widely adopted in the U.S. industry due to the difficulty in cleaning and maintaining them [6].

GAP standards state that insulated windows, solar tubes, or semi-transparent roofing are an acceptable method for providing NL in commercial broiler houses as long as the combined window area is at least 1% of the total floor area and they are placed evenly throughout [7]. Research on light intensity in curtain-sidewall houses providing NL in accordance with GAP standards houses reported significantly higher whole-house mean light intensity (>380 lx) when compared to traditional light-emitting diode (LED) houses providing artificial lighting only (<11 lx) [8]. Temporal sampling over a 24-hr period also revealed increased variations in light intensity throughout the day in the NL design when compared to the traditional LED design [9]. To date, there are no studies available that characterize light intensity in modern commercial houses providing NL via individual windows, which is a more common method of providing NL than lowering sidewall curtains.

In general, there is little guidance on designing broiler houses to provide NL. The ambiguity of GAP NL standards allow for multiple interpretations regarding window size and placement and can lead to drastically different lighting environments within houses. However, the extent to which window size and configuration affect the lighting environment has not been investigated. The objectives of this research were to 1) design and implement a high-density light intensity data collection system and 2) compare light intensity, spatial distribution, and uniformity during brood and tunnel conditions in two GAP-certified commercial broiler houses with different window sizes and configurations.

It is important to note that this research was performed in commercial broiler houses located in the U.S., where many of the larger broiler companies do not build broiler houses that provide NL. Most are constructed with solid sidewalls (no windows) and are tunnel ventilated. Globally, however, broiler chickens are raised using a variety of production methods that include everything from solid-sidewall tunnel ventilated houses to NL provided by windows or dropped curtains to complete outdoor access. Since the U.S. broiler industry has only recently entertained the use of NL in commercial houses, the purpose of this research was to fill the research gaps related to the influence of different window configurations on the lighting environment in U.S. style commercial broilers houses that have been primarily designed as solid sidewall tunnel ventilated houses. Since the goal was to solely characterize light intensities, broiler performance or welfare data is not presented in this manuscript.

## Materials and methods

### House lighting designs

**Fig 1:**
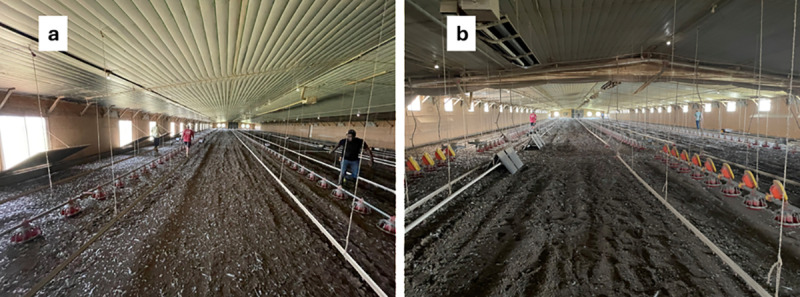
One-sided window (1SW, a) and two-sided window designs (2SW, b) used in study.

Light intensity was assessed in two GAP-certified 18.3 × 183.9 m commercial broiler houses on the same farm in southeast Alabama. Both houses were oriented with tunnel ventilation fans towards the east and the evaporative cooling pads towards the west. The farm consisted of four houses total; the two interior houses were tested to mitigate possible edge effects. One house (Fig 1a) had twenty-three 1.42 × 1.09 m windows only on the north sidewall. The windows had solid single-panel shutters that opened downwards via a hinge on the bottom of the windows. This house served as the one-sided window (**1SW**) design. The other house located 18.9 m north of the first, had fifty-eight 0.95 × 0.60 m windows installed on both sidewalls as well as two same-sized windows on the end wall on the pad end (west wall), all of which had solid single-panel shutters that opened up via a hinge on the top of the windows (Fig 1b). This house will be referred to as the two-sided window (**2SW**) design. Artificial lights were on during testing of brooding and tunnel conditions to simulate the integrator’s lighting program. Target intensities during brooding and tunnel were 43 lx and 1 lx, respectively. More details about the farm and houses can be found in Table 1.

**Table 1:** House and equipment information for design houses.

House Information	1SW Design	2SW Design
House size	18.3 × 183.9 m	18.3 × 183.9 m
Orientation	East – West	East – West
Window size	1.42 × 1.09 m	0.95 × 0.60 m
Number of windows	23	60
Total window area	39.6 m^2^	34.2 m^2^
Tunnel fans	Endwall – 4 Fans	Endwall – 4 Fans
	Sidewalls – 7 and 6 Fans	Sidewalls – 7 and 6 Fans
Fan size	144.8 cm	144.8 cm
Pad length	38.1 m	38.1 m
Rows of lamps	3	3
Lamp spacing	4.9 m	4.9 m
Lamp type	LED/ 6W/ 5000K/ 545 lumens	LED/ 6W/ 5000K/ 545 lumens
Tunnel light intensity set point	1 lx	1 lx
Brood light intensity set point	43 lx	43 lx

### Data acquisition system design and construction

Data was collected on June 28th, 2023, ± 1 h of solar noon as determined by the National Oceanic and Atmospheric Administration Solar Noon Calculator [10]. Solar noon is the time at which the sun is at its highest point in the sky for a specific geographic location. The sampling window around solar noon was chosen to standardize as much as possible the position of the sun relative to the broiler houses. This methodology is most useful when measuring light intensity across multiple days.

**Fig 2:**
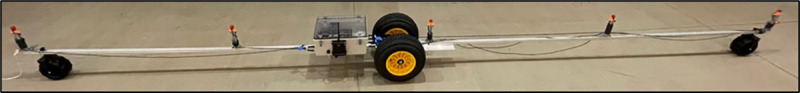
One of the five data collection carts used to collect high-density light intensity data. Five photometric sensors shown with red covers were spaced 1.22 m apart.

Light intensity was measured by a high-density light intensity data collection system (Fig 2) [6]. The system was comprised of five rolling carts made of two 3.05 m long aluminum rails (1515-LS series; 80/10 Inc., Columbia City, IN) and measured a total of 6.1 m in length. Each cart had five photometric sensors (210R; LI-COR, Lincoln, NE) spaced 1.22 m apart. Sensor spacing was designed to match the house truss spacing which was also 1.22 m. Sensor height was 0.46 m to approximate head height of a mature broiler.

Low-amplitude signals from the photometric sensors were transmitted through a universal transconductance amplifier (UTA, EME Systems, Berkeley, CA) connected to a datalogger (CR800; Campbell Scientific, Logan, UT). Radio frequency transmitters and receivers (Viper S-4; RF Solutions, West Sussex, UK) were used to simultaneously collect data from all five carts at the push of a button. The carts were also equipped with wireless network link interfaces (NL241; Campbell Scientific, Logan, UT) to allow for wireless datalogger programming and data retrieval. A 6.1 m pull rope was attached to the front of the carts to reduce the likelihood of operators shading the sensors while collecting data.

### Light intensity data collection

**Fig 3:**
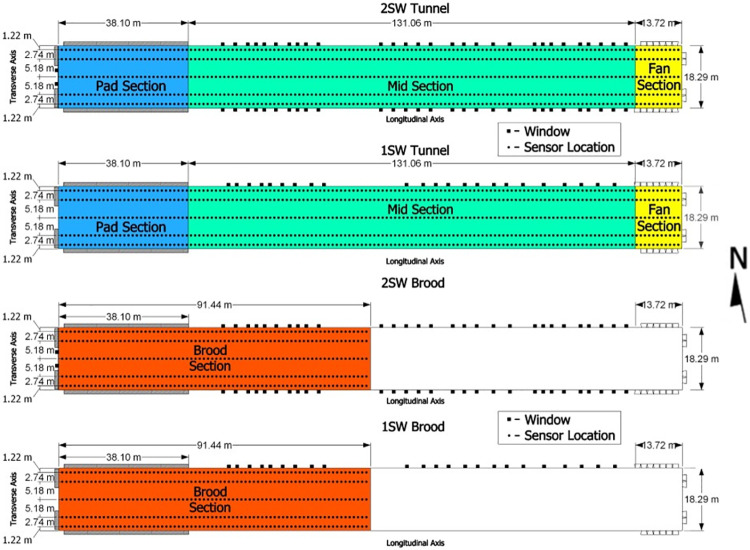
Sensor locations (black dots) and house sections for tunnel and brood conditions in both the 1SW (bottom) and 2SW (top) designs (drawn to scale).

Light intensity data was collected in a high-density grid that consisted of 375 brood and 750 tunnel locations, similar to that described in previous studies [6,8,9,11,12]. Fig 3 shows the sensor positions during data collection for both tunnel and brooding conditions. Since the grower brooded in half the house, only those locations were evaluated for brooding conditions. The carts were spaced 1.2, 4.0, 9.1, 14.3, and 17.1 m from the north wall across the transverse axis (Fig 3). These locations aligned with the lanes of open area between the feed and water lines. The measurement spacing along the length of the houses (longitudinal axis) corresponded with the truss spacing in the houses (1.22 m). Each cart collected 75 brood condition and 150 tunnel condition intensity measurements along the longitudinal axis per collection pass (n = 2). A collection pass as defined as one complete traverse through the houses with all carts. Total light intensity values collected in brood conditions were 750 (5 carts × 75 locations × 2 reps) and 1,500 for tunnel conditions (5 carts × 150 locations × 2 reps). Light intensity data was recorded every second for a ten-second interval at each sampling location. The data can be found in its entirety in the S1 and S2 datasets.

For analysis purposes, the tunnel condition data were segmented into sections: pad section, mid- section, and fan section (Fig 3) as well as whole-house (all sections together). The pad section (155 measurements per collection pass) extended longitudinally from the west end wall to the end of the evaporative pads (38.1 m). The mid-section (535 measurements per collection pass) was considered the area between the pad section and the first tunnel ventilation fan furthest from the east endwall (131.1 m). The fan section (60 measurements per collection pass) extended 16.8 m from the east end wall to the end of the fan bank). The brooding area of each house was considered a single area and was not subdivided. Unlike during tunnel conditions, distinct lighting sections were not apparent in brooding conditions as a result of the elevated target intensity (43 lx). In both the 1SW and 2SW designs, windows were located in the back half of the brood section and throughout the mid-section. No windows were located in the fan section.

### Statistical analyses

Spatial analysis via kriging was performed using the GSTAT package in RStudio [13]. Kriging is a geostatistical interpolation technique used to estimate values at unsampled locations within a spatial domain or grid based on the spatial correlation of observed data points. The size of the grid cells within the spatial domain was 0.3 × 0.3 m, resulting in 36,000 (tunnel) and 18,000 (brood) total predicted data points per house. Fifty-four combinations of predictive models were tested, and it was found that universal kriging with a bessel semi-variogram of log-transformed light intensity performed best. Fivefold leave one out cross validation was used to determine the kriging model performance parameters, including coefficient of determination (r2), mean absolute error (MAE), and root mean squared error (RMSE). Light intensity values were then back transformed for representation in the maps. Coefficient of variation (CV), a standardized measure of dispersion, was calculated for each house section (pad, mid, fan), as well as the whole house. CV is unitless and is therefore useful in comparing variability across datasets with different scales, as was the case with the 1SW and 2SW light intensity datasets.

## Results and discussion

### Tunnel ventilation conditions

Mean light intensity and CV data is presented in Table 2. Whole-house mean light intensity in the 2SW design was higher than the 1SW design (33.0 lx vs 28.3 lx, respectively) despite having 15.7% less total window area. Mean light intensity was also higher in the pad section for the 2SW design than the 1SW (9.7 lx vs 1.4 lx, respectively). This more than 7-fold mean light intensity increase within the pad section was largely due to the two windows located on the end wall in the pad section of the 2SW design.

**Table 2:** Mean light intensity and CV at pad, mid, and fan sections during tunnel ventilation for GAP-certified commercial broiler houses using windows to provide NL through two window designs.

Design	Pad^2^	Mid^3^	Fan^4^	Whole House^5^
Mean Light Intensity (lx)^1^				
1SW	1.4	23.5	140.5	28.3
2SW	9.7	27.9	139.5	33.0
CV (%)				
1SW	39.3	136.5	81.7	192.2
2SW	189.3	53.9	80.2	143.9

Abbreviations: 1SW = one sided windowed design, 2SW = two-sided windowed design.

^1^Number of light intensity measurements per design: pad (n = 155), mid (n = 535), fan (n = 60), whole (n = 750).

^2^Pad = Light intensity measurements extending 38.1 m from endwall nearest the evaporative pads.

^3^Mid = Light intensity measurements from termination of pad section to the first tunnel ventilation fan (131.1 m).

^4^Fan = Light intensity measurements extending 16.8 m from the end wall nearest the fan.

^5^Whole House = All house sections included together.

In the mid-section, mean light intensity in the 2SW design was higher than in the 1SW design (27.9 lx vs 23.5 lx, respectively). At the time of the sampling event, the sun was positioned slightly southwest of the farm. The additional light entering through the south facing windows of the 2SW houses led to the increased mean light intensity values, especially since there were no windows on the south facing sidewall of the 1SW design. Another study that measured light intensity in a commercial house meeting GAP lighting specifications showed a mean intensity level in the mid-section of 77.1 lx, which is higher than both designs presented here [9]. However, the houses measured were curtain-sided and did not have individual windows like the houses tested in this study. NL was provided by dropping the existing curtain and covering the opening with a translucent curtain. Differences in intensity between these studies could be attributed to a number of factors, including geographic location, time of day, cloud cover, season, or lamp intensity.

Mean light intensity in the fan sections were close in value due to the similar configurations of tunnel ventilation fans across the two houses. Within each window design, the fan sections displayed higher light intensity levels than the pad and mid sections. The fan section mean light intensity in the 1SW design was 6 and 104 times higher than the mid and pad sections, respectively, while the 2SW design was 5 and 14.4 times higher than the mid and pad sections, respectively. These findings are similar to those presented in previous studies [2,5,6,8,9]. Interestingly, even with windows installed in the mid-section of each house, the fan section still experiences much higher light intensities. This is most likely caused by the individual opening created by a fan being larger than that created by the windows and because the fans are concentrated in one area of the house and not spread out as much on the sidewalls as the windows. Individual fan openings were 8.5% and 187.7% larger than window openings in the 1SW and 2SW houses, respectively. Additionally, the windows were dirty and depreciated some of the light entering through them. Spinning fan blades most likely lead to some light depreciation, but upon general observation, it was less than the windows.

The CV of the mid-section was more than 2.5 times higher for the 1SW design when compared to the 2SW design mid-section (1SW mid-section CV = 136.5% vs 2SW mid-section CV = 53.9%) (Table 2). In fact, all the CVs were higher for the 1SW design except for the pad section indicating that the 1SW design led to a decrease in light uniformity. The highest uniformity was found to be in the pad section of the 1SW design (39.3%), which most closely resembled the light environment in a house without the provision of NL due to the lack of windows.

**Fig 4:**
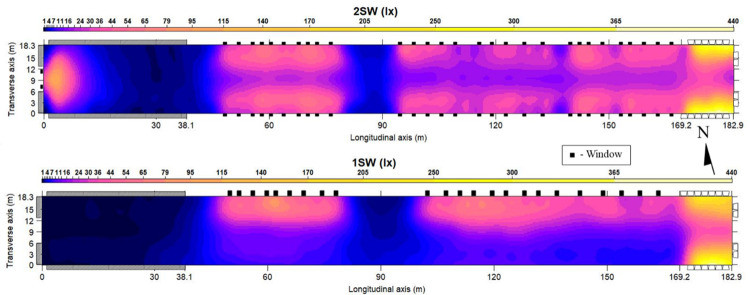
Spatial light intensity distribution maps of 1SW and 2SW design houses during tunnel conditions. Size of windows (black boxes) are to scale for comparison. Target light intensity for artificial lights during tunnel conditions was 1 lx.

Light intensity maps of tunnel conditions for both designs are displayed in Fig 4. Notably, the pad section of the 2SW design was heavily influenced by the two windows in the end wall. Perhaps the most obvious difference between the designs is the variation of light intensity across the transverse axis. For example, within the mid-section, the 1SW design had higher intensities along the north sidewall where the windows were located, compared to the south sidewall. The 2SW design did not have such a contrast in intensities between sidewalls, with intensities being similar between the north and south sidewalls. This is supported by the 1SW mid section’s CV of 136.5%, which is considerably higher than the 2SW mid-section CV of 53.9%. Whole-house CVs were higher in both the 1SW (192.2%) and 2SW (143.9%) designs of the present study compared to the NL design (119.1%) created by lowering a curtain [9].

Table 3 shows the fit statistics for the kriging maps presented in Fig 4. The r^2^ value for the 2SW design was higher than for the 1SW design indicating that the 2SW kriging model better fit the measured data. The reason for this is due to the previously mentioned window shutters that opened down in the 1SW design, shading the floor directly below. This created localized areas of high and low light intensity and while kriging does a good job predicting trends over larger areas, the technique does not always perform as well at predicting drastic changes in values within localized areas. The RMSE is also larger for the 1SW house than the 2SW house because it is more sensitive to the larger errors created by the 1SW window design. With RMSE, the errors are squared before averaging which gives more weight to larger errors. The ratio of RMSE to the range of values for the 1SW and 2SW designs were 6.0 and 2.9% percent, respectively, which indicates a generally acceptable model fit. Since MAE is the average of the differences between the model predictions and the measured data at given locations, an overall positive ME indicates that the kriging model slightly overpredicted light intensities for both the 1SW and 2SW designs (10.2 lx and 4.6 lx, respectively). For the 1SW design, most of the overprediction occurred near the fan section and in the mid-section of the house near the windows. In the 2SW house, overprediction mainly occurred near the fans. As mentioned earlier, kriging errors can be magnified in areas where there are drastic changes in values within localized areas, as was the case in the 1SW house near the windows and in both houses near the fan section where light intensities were higher than the rest of the house.

**Table 3:** Kriging cross validation and fit statistics for 1SW and 2SW designs during tunnel conditions.

Statistic	1SW	2SW
MAE (lx)	10.2	4.6
RMSE (lx)	26.5	12.9
r^2^	0.77	0.93

### Brooding conditions

Mean light intensities in the 1SW and 2SW designs (44.7 lx and 43.7 lx, respectively) were very similar to artificial light set point levels (43 lx) (Table 4). Unlike during tunnel conditions, the higher intensities from the artificial bulbs seemed to partially mask the effects of the windows. Similar to the CV’s for tunnel condition data, the CV of the brood condition data in the 1SW design was higher than the 2SW design (1SW brood CV = 63.7% vs 2SW brood CV = 56.4%), indicating less spatial unformity int the 1SW design. However, this difference in uniformity between designs was less pronounced than during tunnel conditions due to masking effect of the brighter artificial lights (brooding target = 43 lx; tunnel target = 1 lx).

**Table 4:** Mean light intensity and CV at brood section during brood conditions for GAP-certified commercial broiler houses using windows to provide NL through two window designs.

Design	Mean light intensity (lx)	CV (%)
1SW	44.7	63.7
2SW	43.7	56.4

**Fig 5:**
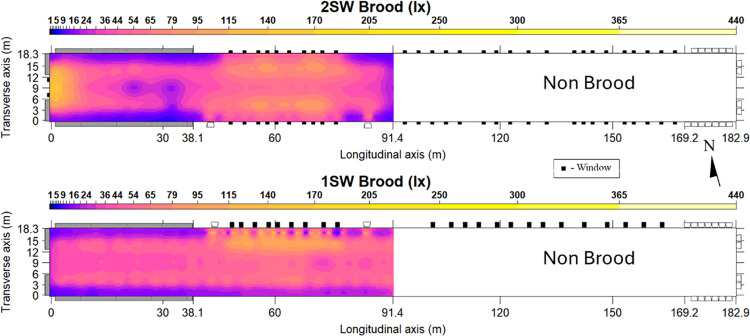
Spatial light intensity distribution maps of 1SW and 2SW designs during brood conditions. Size of windows (black boxes) are to scale for comparison. Target light intensity for artificial lights during tunnel conditions was 43 lx.

Maps of light intensity for both designs during brood conditions are found in Fig 5. The shading effect of the window shutters was evident in the 1SW design brood map, whereas this effect was absent in the 2SW design. The difference can be attributed to the design of the window shutters in the 2SW design, which open upward instead of downward, as in the 1SW design. Consequently, this design prevented them from shading the floor in the 2SW design. Table 5 shows the fit statistics for the kriging maps presented in Fig 5. As with the tunnel condition maps, the r^2^ value for the 1SW design was lower as a result of the shading created by the shutters and the model slightly overpredicted for each design as indicated by the MAE. The ratio of RMSE to the range of values for the 1SW and 2SW designs were 1.6 and 1.4% percent, respectively, which indicates a generally acceptable model fit. Since influence the from the fans was not present during brooding conditions to increase model errors, RMSE values were better for brooding conditions than tunnel conditions.

**Table 5:** Kriging cross validation and fit statistics for 1SW and 2SW designs during brooding conditions.

Statistic	1SW	2SW
MAE (lx)	9.5	4.2
RMSE (lx)	6.1	5.9
r^2^	0.89	0.95

### Intensity profiles in windowed areas (mid-section)

**Fig 6:**
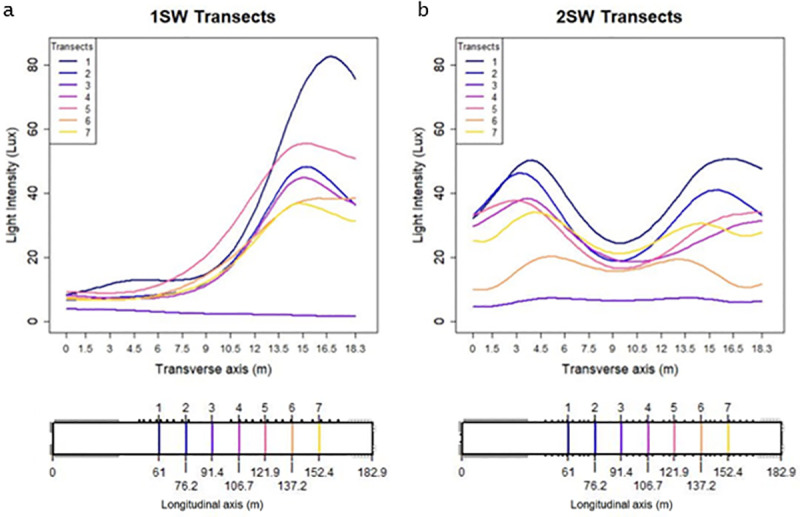
Line graphs of transects taken from kriged maps showing change in light intensity along the transverse axis of the 1SW (a) and 2SW (b) designs. A (lower) and b (lower) show location of transects along the longitudinal axis of the 1SW and 2SW designs, respectively.

Fig 6a, b show a collection of seven transects in the transverse direction taken from the kriged data at 61, 76.2, 91.4, 107.6, 121.9, 137.2, and 152.4 m along the longitudinal axis for the 1SW and 2SW houses, respectively. It is clear that two very distinct light intensity profiles are created by the 1SW and 2SW designs. Light ingress from the windows on both sides of the 2SW house create areas of elevated light intensity near the windows with a peak at 3–4 m from the sidewalls. The lowest light intensities are found along the floor near the ceiling peak. The 1SW house also has the same elevated light intensities but only near the sidewall with the windows. Light intensities near the ceiling peak are only slightly higher than those found near the sidewall without windows.

The transects also demonstrate that the light intensity profiles vary substantially depending on the location along the longitudinal axis and is largely dependent on window spacing. For example, profiles near the center of the house have the lowest intensities, primarily because there was a larger distance between windows. The center of the house was where the control room, brood curtain, and feed hoppers are located and was, therefore, a difficult place to install windows. Variation in intensity between the profiles were largely influenced by window spacing with higher intensities in areas where the windows are closer together.

This study shows that different window configurations in commercial broiler houses that both meet GAP standards can result in different lighting environments. GAP provides little design guidance in its standards and has largely left the specifics, such as window size and placement, up to the integrators. Consistent light intensities and photoperiods (daylengths) are valued by the industry in traditional, non-windowed houses so that birds in different areas of the house have similar growing environments. Although past research [2,5,6,8,9] has shown that the lighting environment can be varied even in traditional, non-windowed houses primarily as a result of operating ventilation fans, variation in light intensities in NL houses is more pronounced and likely to be influenced by external conditions (cloud cover, seasons, house orientation). Although the internal environments of NL houses are susceptible to changes in external conditions, this research shows that design considerations can be taken into account when installing windows. For example, windows placed as evenly as the house will allow on both sidewalls will yield better overall uniformity. In addition, window shutters that open upwards will alleviate shaded areas created by downward opening shutters and will allow the windows to remain open in between flocks because they will not get in the way of equipment.

In the larger context of broiler production, the influence of NL via windows is not limited to varied light intensities as presented here. Unpublished data collected by the authors shows that birds in a commercial house providing natural light were 96 g lighter and had a higher feed conversion ratio (FCR) than those in a traditional LED house (1.548 vs 1.495, respectively). Lighter birds and increased FCR can lead to reduced grower and company profits. In addition, it is a known fact that the addition of windows to any building increases heat loss/gain. However, when large-scale production changes are made by companies in response to shifting consumer and retailer demands, the effect of these changes is often shouldered by the grower as higher energy and fuel costs and other building-related expenditures. Thus, there is a need to understand the influence of NL in the larger context of broiler production, which includes grower costs, company profits, and environmental impacts from fuel, electricity, and water consumption. Research efforts that attempt to understand the balance between bird welfare and the overall sustainability and profitability of the industry are warranted.

## Conclusions

1. Though both window designs meet GAP NL standards, the light environments differ in both intensity and uniformity due to alternate window configurations. Window placement and shutter design greatly influence the spatial distribution of light intensity.
2. During tunnel conditions, the 2SW design created brighter light intensities in the mid and whole-house sections than the 1SW.
3. The 2SW design exhibited better light uniformity in the mid and whole-house sections than in the 1SW design during tunnel conditions.
4. Brooding conditions were more similar between the designs because the higher artificial light target intensity (43 lx) decreased the variation in NL across the floor area.
5. Tunnel ventilation fans, when in operation, led to higher light intensities than the windows.

## Supporting information

S1 Dataset Light intensities and house measurement coordinates for during tunnel conditions. (XLSX)

S2 Dataset Light intensities and house measurement coordinates for brooding conditions. (XLSX)
